# Carbon ion radiotherapy using fiducial markers for prostate cancer in Osaka HIMAK: Treatment planning

**DOI:** 10.1002/acm2.13376

**Published:** 2021-08-02

**Authors:** Toshiro Tsubouchi, Noriaki Hamatani, Masaaki Takashina, Yushi Wakisaka, Atsuhiro Ogawa, Masashi Yagi, Ayumi Terasawa, Kazuyuki Shimazaki, Masashi Chatani, Jun’etsu Mizoe, Tatsuaki Kanai

**Affiliations:** ^1^ Osaka Heavy Ion Therapy Center Osaka City Osaka Japan; ^2^ Department of Carbon Ion Radiotherapy Osaka University Graduate School of Medicine Suita City Osaka Japan; ^3^ Hokkaido Ohno Memorial Hospital Sapporo Hokkaido Japan

**Keywords:** beam‐specific planning target volume (bsPTV), carbon ions, Osaka HIMAK

## Abstract

**Purpose:**

Carbon ion radiotherapy for prostate cancer was performed using two fine needle Gold Anchor (GA) markers for patient position verification in Osaka Heavy Ion Medical Accelerator in Kansai (Osaka HIMAK). The present study examined treatment plans for prostate cases using beam‐specific planning target volume (bsPTV) based on the effect of the markers on dose distribution and analysis of target movements.

**Materials and Methods:**

Gafchromic EBT3 film was used to measure dose perturbations caused by markers. First, the relationships between the irradiated film density and absolute dose with different linear energy transfer distributions within a spread‐out Bragg peak (SOBP) were confirmed. Then, to derive the effect of markers, two types of markers, including GA, were placed at the proximal, center, and distal depths within the same SOBP, and dose distributions behind the markers were measured using the films. The amount of internal motion of prostate was derived from irradiation results and analyzed to determine the margins of the bsPTV.

**Results:**

The linearity of the film densities against absolute doses was constant within the SOBP and the amount of dose perturbations caused by the markers was quantitatively estimated from the film densities. The dose perturbation close behind the markers was smallest (<10% among depths within the SOBP regardless of types of markers) and increased with depth. The effect of two types of GAs on dose distributions was small and could be ignored in the treatment planning. Based on the analysis results of internal motions of prostate, required margins of the bsPTV were found to be 8, 7, and 7 mm in left–right (LR), anterior–posterior (AP), and superior–inferior (SI) directions, respectively.

**Conclusion:**

We evaluated the dose reductions caused by markers and determined the margins of the bsPTV, which was applied to the treatment using fiducial markers, using the analysis results of prostate movements.

## 1 INTRODUCTION

Carbon ions have a different dose distribution from X‐rays, known as the Bragg peak, which gives a large dose at the end of range, as well as a higher biological effect than X‐rays, leading to high dose constraints to targets and less damage to normal tissues.^1^

Treatment of patients with carbon ion radiotherapy (CIRT) at Osaka Heavy Ion Medical Accelerator in Kansai (Osaka HIMAK) began in October 2018. The center has three treatment rooms, each with two fixed ports. Orthogonal X‐ray imaging systems for verification of patient positions are installed at all treatment rooms. A computed tomography (CT) scanner can be used in treatment room 2 for three‐dimensional (3D) image registration. Carbon ions are delivered using a raster scanning method with a maximum field size of 20 × 20 cm^2^. There are 12 accelerated energies from 100 to 430 MeV/u, and 100 energies can be produced by combining with range shifters. The interval of each energy is equivalent to 3 mm of water equivalent thickness. The VQA treatment planning system (Hitachi, Ltd.) is used at Osaka HIMAK and the Kanai model has been adopted for deriving the biological dose.^2^, ^3^

The treatment schedule of 12 fractions over 3 weeks for prostate cancer^4^ is adopted by our center. The prescription dose of 51.6 Gy (RBE) in 12 fractions is delivered with parallel‐opposed lateral fields.^4^

It is known that bone matching is only performed as patient position verification at CIRT facilities in Japan and planning target volume (PTV) margins are set large enough to cover prostate with considering uncertainties due to set up errors and internal motions of the prostate during treatment.^4^, ^5^ On the other hand, marker matching as position verification makes it possible to accurately deliver dose to a clinical target volume (CTV) and optimize margins for covering the CTV. To operate the marker matching in clinical use, we applied the beam‐specific PTV (bsPTV),^6^, ^7^ which can compensate range uncertainties caused by setup errors and internal motions. Gold Anchor (GA, Naslund Medical AB) is adopted at our center as a tool of patient setup verification in prostate cases.^8^ It is reliable and provides accurate position verification, resulting in high‐precision dose delivery to the prostate.^9^, ^10^

The present study evaluated the effect of two types of fiducial markers, GA and VISICOIL (RadioMed), on dose distributions measured by films. Several studies evaluating the dose perturbation due to fiducial markers for proton beams have been reported^10^, ^11^, ^12^, ^13^, ^14^, ^15^; however, only one study for carbon ion beams has been reported.^16^ Treatment plans with the bsPTV for prostate cancer are created using single‐field uniform dose (SFUD) algorithm since this algorithm is more robust against uncertainties than intensity‐modulated particle therapy (IMPT).^17^

More than 1000 fractions have already been delivered to prostate cancers at Osaka HIMAK. The present study reports on the treatment plans created by SFUD using the bsPTV based on the investigation of the effect of markers placed in the prostate on the dose distribution as well as the internal motions of the prostate derived from irradiation results.

## 2 METHODS

This study was approved by institutional review board.

### 2.1 Measurement of dose perturbation caused by markers

#### 2.1.1 Experimental conditions

Gafchromic EBT3 radiochromic film was used to measure dose distribution.^18^, ^19^ Radiochromic film has linear energy transfer (LET) dependence, which means that the dose response of the film to radiation with high LET is low due to the quenching effect.^20^, ^21^ It is not possible to directly convert the optical density of films irradiated by carbon ions into absolute doses without any processing because carbon ion beams have widely distributed LET values, ranging from a few keV/μm to >100 keV/μm. However, dose evaluation using the film can be performed by confirming the relationship between irradiated doses and film densities in specific irradiation conditions, which are used for the measurement of dose distributions behind markers based on the assumption that the lateral LET distributions at the same depth are constant and unchanged. Coulomb scattering by markers is expected to lead to fluence reduction and consequent dose reduction.

**FIGURE 1:**
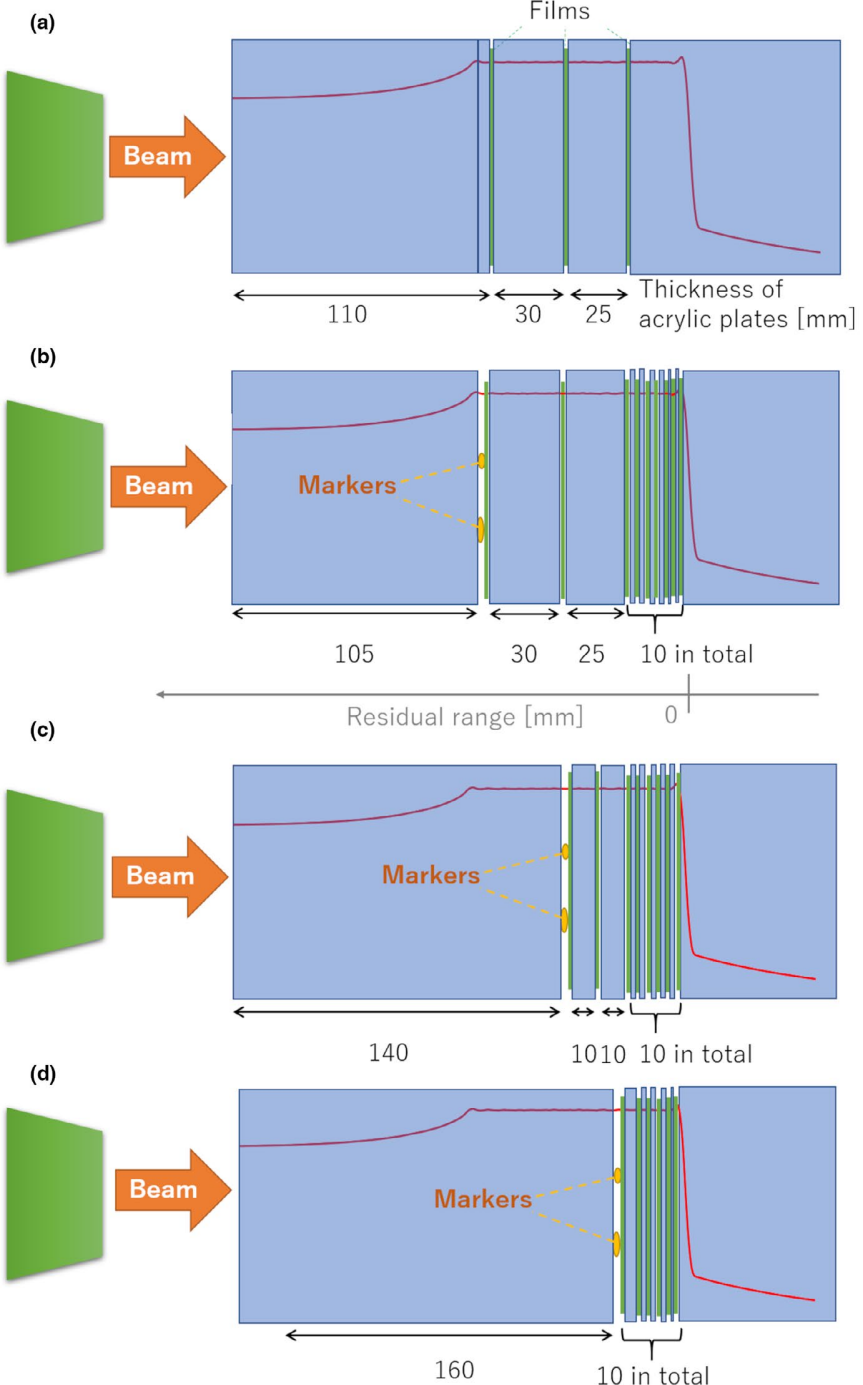
Experimental setups. (a) Three films were set between acrylic plates to measure the doses at three different depths with different LET distributions. Markers were installed at (b) proximal, (c) center, and (d) distal depths in the SOBP and the films were set behind the markers to measure dose perturbations caused by the markers. LET, linear energy transfer; SOBP, spread‐out Bragg peak

Carbon ion beams were delivered in the range of 20.0 and 8.0 cm of spread‐out Bragg peak (SOBP), in which the physical dose was constant. First, to determine the relation of delivered doses with optical densities of the films within the SOBP, films were set at proximal, center, and distal depths within the SOBP and irradiation was performed, changing the dose 0.5–2.0 times. The measurement condition is summarized in Figure 1a. Four types of markers, GA [*φ*, 0.28 mm, length, 10 (short), and 20 (long) mm, respectively] and VISICOIL [length, 5 mm, *φ*, 0.5 (small), and 0.75 (large) mm, respectively], were used to evaluate the dose perturbations caused by them. The same irradiation condition was used. The markers were placed at the proximal, center, and distal depths within the SOBP, and the dose distributions were measured by films, which were installed behind the markers between acrylic plates at different depths (Figure 1b–d).

By fitting the data of dose perturbation measured by EBT3 film with a defined function, the ratio of dose reduction to the delivered dose (dose reduction rate) and the region caused by the markers within the SOBP (target) can be derived. To show the effect of markers on dose distributions in the clinical case, a dose‐volume histogram (DVH) on a virtual target, in which the markers were set at proximal, center, and distal depth, respectively, was calculated from the derived data. In the simulation, the target volume was set to 32 cm^3^, which was equivalent to the typical prostate volume, and the maximum depth and target length along depth were set to the same values (20 cm and 8 cm) as in the experimental condition to apply the experimental results. Although the field size is small (2 × 2 cm^2^), the dose reduction is overestimated.

#### 2.1.2 Film analysis

Each film was scanned using a resolution of 150 dpi on a flatbed scanner (DS‐G20000, Seiko Epson Corp.) prior to irradiation to obtain a background value. The irradiated films were then scanned using the same resolution 24 h after irradiation, and the images obtained were saved as uncompressed tagged image files. The red channel was extracted from the RGB transmission image and used for analysis.

The film density (optical density; OD) was derived from the extracted red channel as follows^21^:

(1) $$\text{netOD}={\text{OD}}_{\text{irr}.}‐{\text{OD}}_{\text{BG}}=‐\log\left[\frac{{\text{PVR}}_{\text{irr}.}}{{\text{PVR}}_{\text{BG}}}\right]$$

where PVR_irr_ and PVR_BG_ represent the red channel's value of films with and without irradiation, respectively, and the mean values of red channel's values in the square region of interest were used.

### 2.2 Interfractional and intrafractional motion of prostates

Two types of vacuum cushions (VacQfix and BlueBAG BodyFIX vacuum cushions) are used at Osaka HIMAK as an immobilization tool for prostate cancer patients. Fiducial marker‐based patient position verification is performed using orthogonal X‐ray systems, which provides front and side images of the patients. First, bone matching using pubis and pubis symphysis is performed with the tolerance of 0.5 mm and 0.1 degree below which residual motion can be ignored due to bone displacement. Next, marker matching that allows only translation to markers is performed, and the translation is regarded as interfractional motion of the prostate. In the initial three fractions, X‐ray images are acquired even after irradiation and the marker displacements before and after irradiation are recorded for each patient to evaluate intrafractional motion of the prostate. The present study used data from 100 patients (from July to October 2019) to derive the intrafractional and interfractional motions.

### 2.3 Treatment planning

#### 2.3.1 bsPTV

Unlike gross tumor volume and CTV, the PTV depends on the type of radiation delivered. For CIRT, anatomical changes induce range variations and have a significant effect on dose distribution. The bsPTV, which needs to be created for each beam, can provide a robust dose distribution against such variations. The bsPTV is designed as follows.^6^ First, a CTV is laterally expanded with a margin (hereafter, bs‐lateral margin) to encompass internal motions of a target and setup errors. Second, ray tracing is performed to calculate the radiological path length from the patient's surface to distal or proximal surface of the expanded CTV. Third, margins accounting for systematic range uncertainties are added for each ray.^22^ Fourth, the calculated radiological path length at a certain point is replaced with the maximum (minimum) length for distal (proximal) surface within the bs‐lateral margin from that point to avoid inhomogeneity of dose distributions due to patient anatomical changes along the beam path. The calculation above is performed by converting all voxels on a beam path to water equivalent. Therefore, in the final step, the margins calculated in the water equivalent representation are converted to the geometrical ones from the density of each voxel calculated from the CT values. Furthermore, a virtual collimator, which is a function in VQA, is applied to specify the irradiation field size. The margin in the present study was defined using the equation:

(2) $$\mathrm{Margin}=\sqrt{\begin{array}{c} Inter^{2}+Intra^{2}+Contouring^{2}+Positioning^{2}+Beam\;Axis^{2}\\ +Stopping\;Power^{2}+Sys\;Range^{2} \end{array}}$$

where *Inter* and *Intra* represent the motion of targets, the uncertainty of delineating targets is included in *Contouring*, *Positioning* includes random errors due to therapists’ techniques and systematic errors from the patient position verification system, *Beam Axis* represents the deviation of the beam axis and range uncertainty due to the uncertainties of CT images and theoretical calculation of CT values, energy dependency of stopping power is represented as *Stopping Power*, and *Sys Range* represents daily range fluctuations.

Parameters in this equation depend on the direction of the margin to be given. For example, bs‐lateral margin, within which ray tracing is performed, can be determined by excluding the uncertainty of the beam direction (*Stopping Power* and *Sys Range*) in Equation (2). On the other hand, only *Intra*, *Contouring*, *Positioning*, and *Beam Axis* are considered to effectively deliver dose to the CTV since we assume interfractional motion can be compensated by the marker matching.

The shape of the bsPTV that creates robust dose distributions against structural changes along a beam path depends on each beam. However, it is assumed that a geometrical PTV can be used for the evaluation of the dose distribution on the CT images where a treatment planning is created. We define the geometrical PTV to evaluate the dose distribution as PTV_evl_ and the margins of bsPTV and PTV_evl_ in all directions were derived (Table 1).

**TABLE 1:** Margins of bsPTV and PTV_evl_ in LR, SI, and AP directions. To determine the margin derived from stopping power, the depth to the target is set to 200 mm

	bsPTV			PTV_evl_		
	LR [mm]	SI [mm]	AP [mm]	LR [mm]	SI [mm]	AP [mm]
Interfractional motion	1	4.8	4.7	—	—	—
Intrafractional motion	1	2.7	2.9	1	2.7	2.9
Contour	3			3		
Positioning	1			1		
Beam axis	—	1	1		1	1
Stopping power	7 200 × 0.035	—	—	7 200 × 0.035	—	—
Sys range	1	—	—	1	—	—
Total margin based on Equation (2)	7.94	6.43	6.44	7.81	4.28	4.41
**Margin**	**8**	**7**	**7**	**8**	**5**	**5**

The bold value means the calculation results of margins.

### 2.4 Procedure of creating treatment plans

Two GAs in a cylindrical shape are inserted in a prostate, vertical to a beam direction, after which they are folded into a ball shape in the prostate; however, the shape depends on patients and doctor's manipulation. To minimize the effect of markers, it is recommended that two markers are implanted to not overlap each other on the beam path and are placed at an interval within the prostate.

Treatment plans need to include the effect of the markers and address prostate movements. They are created using CT images reconstructed using a metal artifact reduction technique, Single Energy Metal Artifact Reduction (SEMAR, Canon medical systems Ltd.), to reduce artifact generated due to markers. The bs‐lateral margin is set to 10 mm, up to which rigid translation to markers after bone matching is theoretically allowed. This value and irradiation field size need to reflect the amount of inter‐ and intrafractional motion of the prostate, respectively. The margin in the beam direction (LR margin in Table 1) depends on the target depths of each patient. The treatment plans are created to satisfy the criteria of D_95_ ≥ 95% of the prescribed dose for PTV_evl_. The dose constraints for the rectum are V_51.6 Gy (RBE)_ ≤ 0.0 cc, V_46.44 Gy (RBE)_ ≤ 2.6 cc, V_41.28 Gy (RBE)_ ≤ 4.5 cc, V_36.12 Gy (RBE)_ ≤ 6.2 cc, and V_25.8 Gy (RBE)_ ≤ 9.3 cc. These values are based on the rectal dose data from the treatment results of prostates with carbon ion radiotherapy at Gunma University^23^ and they are the recommended dose constraints. The dose constraints for the bladder are V_44.89 Gy (RBE)_ ≤ 25% and V_28.38 Gy (RBE)_ ≤ 45% and the dose constraint for small bowel is set to D_0 cc_ ≤ 40 Gy (RBE) if it is close to an irradiation field.

## 3 RESULTS

### 3.1 Relationship between film density and absolute dose, and dose distribution behind markers

**FIGURE 2:**
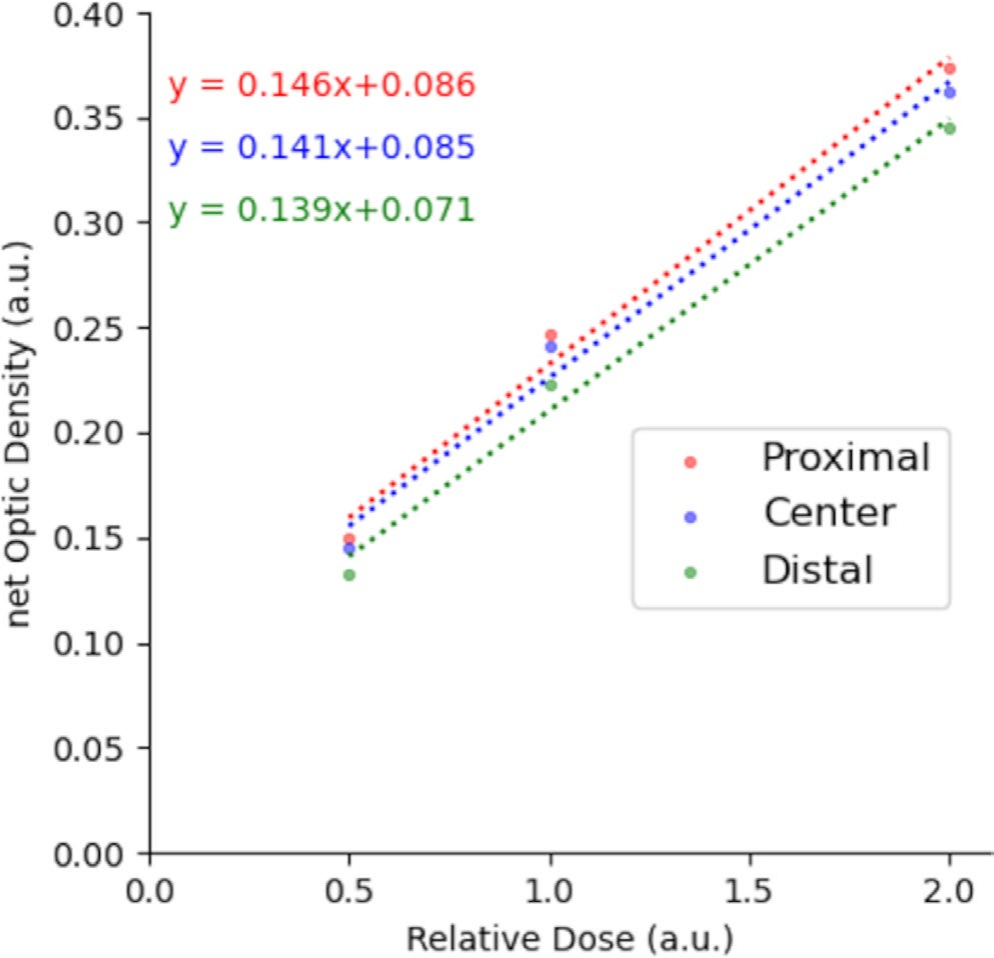
Relationship between film density and physical doses at three depths within a SOBP. Small, medium, and large doses were administered and each dose was normalized to the medium dose. The film density showed linearity with dose regardless of depth, and the linear function for each depth was derived using the least squares method. SOBP, spread‐out Bragg peak

In the present study, the relationships between the film density and absolute dose at different depths within a fixed SOBP were found to be linear and each linear function in Figure 2 was derived using the least squares method, the gradients of which were 0.146, 0.141, and 0.139, respectively. It can be assumed that the relationship of the dose with the film density is expressed by one linear function regardless of the depth. The mean value of all gradients was used to derive the dose perturbation from the film densities independent of depth.

**FIGURE 3:**
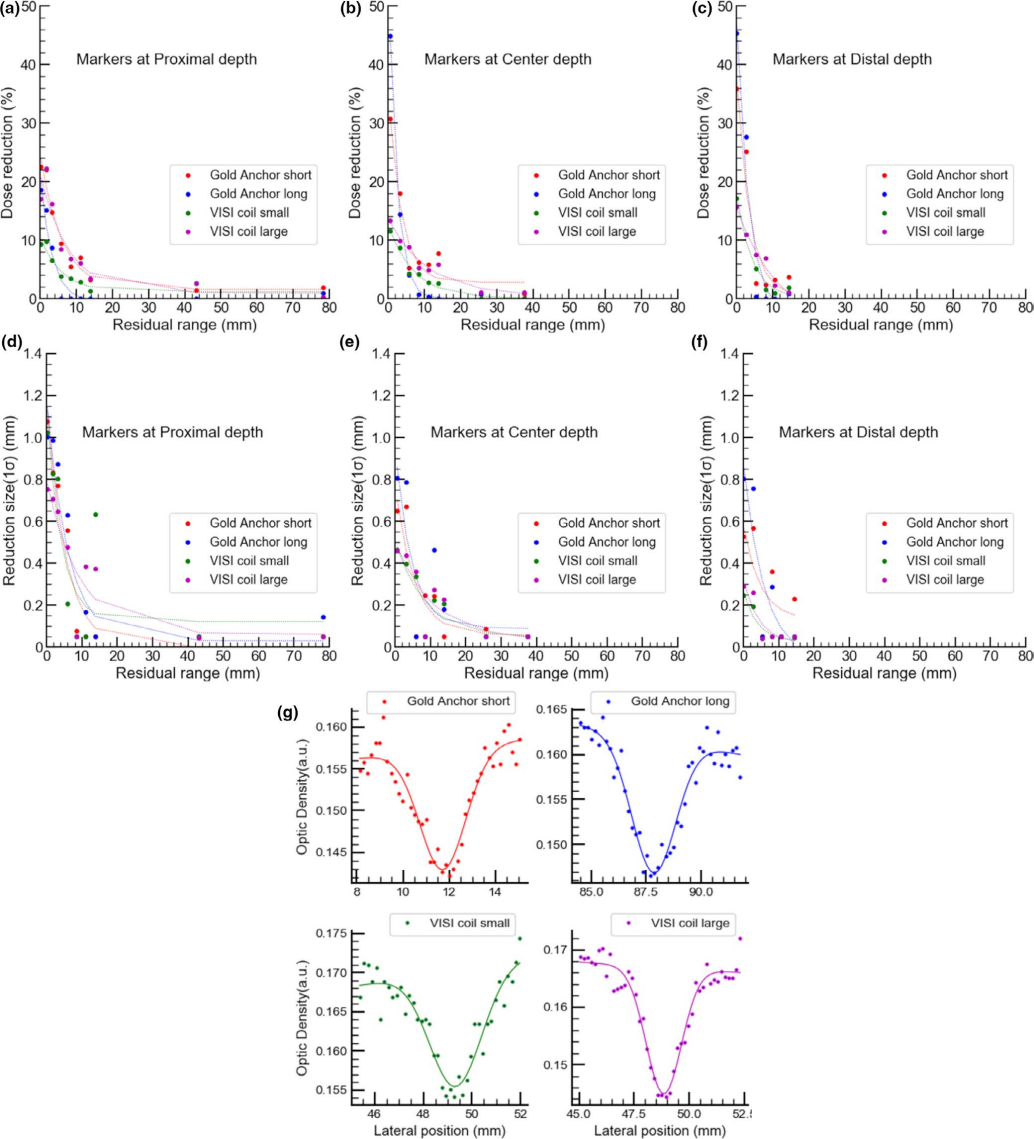
Effect of markers set at different depths on dose distributions. The dose reduction as a function of residual range is shown in (a–c) and the region of dose perturbation due to markers is shown in (d–f), which was derived by fitting measured data with the composite function. (g) shows a fitting result when dose reductions were measured around a residual range of zero and markers were placed at a proximal depth

Dose perturbations due to each marker are shown in Figure 3. The dose reduction rates are shown in Figure 3a–c when the marker was set at (a) proximal, (b) center, and (c) distal depths within the SOBP. Figure 3d–f show the dose reduction regions at (d) proximal, (e) center, and (f) distal depths of markers, which were derived by fitting each measured data with the composite function of Gaussian and linear function (Figure 3g). The dose reduction and the reduction region due to markers were the largest when the depth of the residual range was zero. Up to 50% dose reduction caused by GA (l = 20.0 mm) was confirmed, and the maximum dose perturbation region for both markers was 1*σ* = 1.2 mm.

**FIGURE 4:**
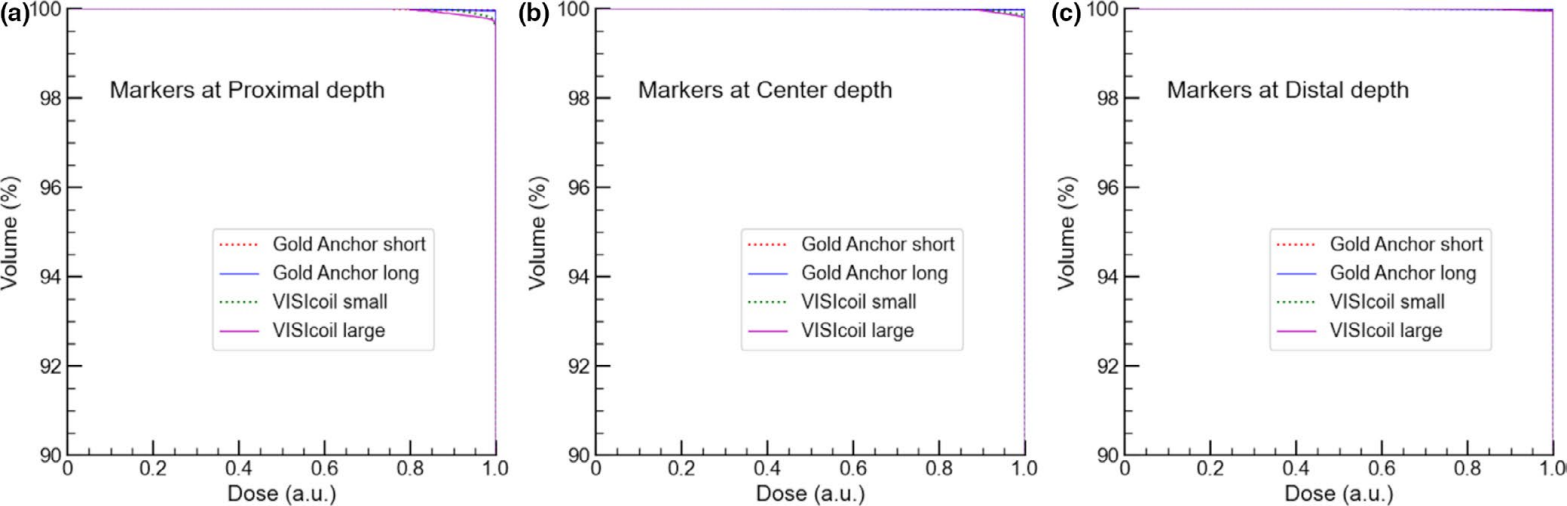
Using the measured data, dose reduction and the region caused by fiducial markers, dose‐volume histograms of a target, inside which the markers were set at (a) proximal, (b) center and (c) distal depth, are simulated

The dose reduction rate and the region represented as a function of residual range were fitted using the following function with the least squares method.

(3) $$y=e^{‐ax+b}+c$$

where *x* represents residual range and *a*, *b*, and *c* are the fitting parameters. They are uniquely determined by each measured data. Using the fitted data, DVHs for both markers at different depths were calculated and shown in Figure 4, indicating that the effects of the markers are very small.

### 3.2 Prostate movement in HIMAK and bsPTV margins

**FIGURE 5:**
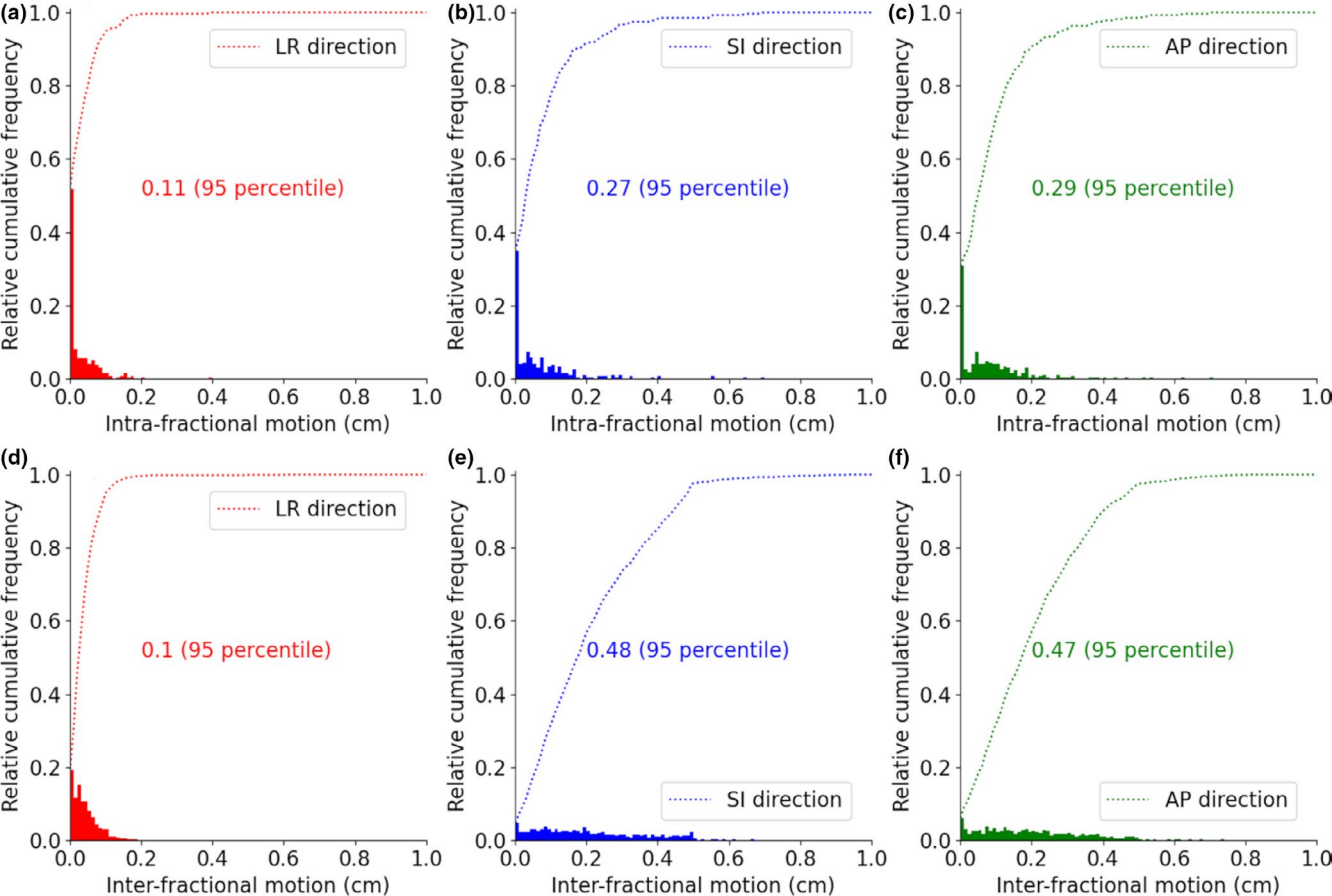
Cumulative distributions of intrafractional motion (a–c) and interfractional motion (d–f) are shown. Numerical values indicate 95th percentile values

The interfractional and intrafractional motions of prostates in each direction are summarized in Figure 5. The interfractional and intrafractional motions of the 95th percentile were 1.0, 4.7, and 4.8 mm, and 1.1, 2.9, and 2.7 mm in left–right (LR), anterior–posterior (AP), and superior–inferior (SI) directions, respectively. The results show that the target movement in LR direction was smaller than that in other directions.

The value of 95th percentile is used to derive the PTV margin. In Osaka HIMAK, *Contour* is set to 3 mm,^24^, ^25^ *Positioning* is set to 1 mm, *Sys Range* is set to 1 mm, and *Stopping Power* is derived by multiplying the depth to the target by 3.5%,^22^ assuming that the depth is 200 mm. The margins of bsPTV and PTV_evl_ were calculated using Equation (2). As shown in Table 1, the required margins of bsPTV and PTV_evl_ were 8.0, 7.0, 7.0, and 8.0, 5.0, 5.0 mm in LR, SI, and AP directions, respectively.

## 4 DISCUSSION

The film response to delivered dose with different LET distributions within a SOBP was first confirmed and showed in similar responses within the SOBP, regardless of the different LET distributions (Figure 2). Based on this finding, the dose reductions caused by GA and VISICOIL folded into a ball shape were estimated from the derived approximation (Figure 3a–c). The cold spots directly behind the markers were relatively small and became largest around the depths of the residual range of zero. The maximum dose reduction region derived by a Gaussian fitting was 1.2 mm. The dose perturbation in the case of carbon ions evaluated in our study is much smaller than those that were reported in the case of protons. This is because multiple coulomb scattering of protons occurs in a greater amount than carbons.^11^, ^12^, ^14^ On the other hand, contribution of fiducial markers to dose distributions of carbon ions is small, which is similar to the results of another study.^16^ In the present study, the DVH of a target was simulated to evaluate the effect of markers on dose coverage to a target. The results indicated that although the dose coverage changes depending on the position and types of markers, both markers, GA and VISICOIL, have a very small impact on the coverage regardless of the position of the markers inside the target, suggesting that the effect of markers can be ignored for dose calculations. Moreover, irradiations with laterally parallel‐opposed beams mitigate that perturbation.^14^

Some particle therapy centers in Japan use a thermoplastic shell as an immobilization tool for prostate cases. In Osaka HIMAK, vacuum cushions (below the upper thigh) are used instead of the thermoplastic shell. Song et Al. reported that use of various immobilization tools showed no significant difference in overall prostate movements, and the amount of interfractional and intrafractional prostate motion in the present study (Figure 5) was comparable with that reported previously.^26^, ^27^, ^28^

The margins of bsPTV and PTV_evl_ were decided as shown in Table 1. There is still room to improve these margins. The margins for intrafractional and interfractional motions can be set with higher reliability using more data about prostate motions when it has accumulated. Moreover, smaller margins can be set using treatment machines with higher accuracy.

**FIGURE 6:**
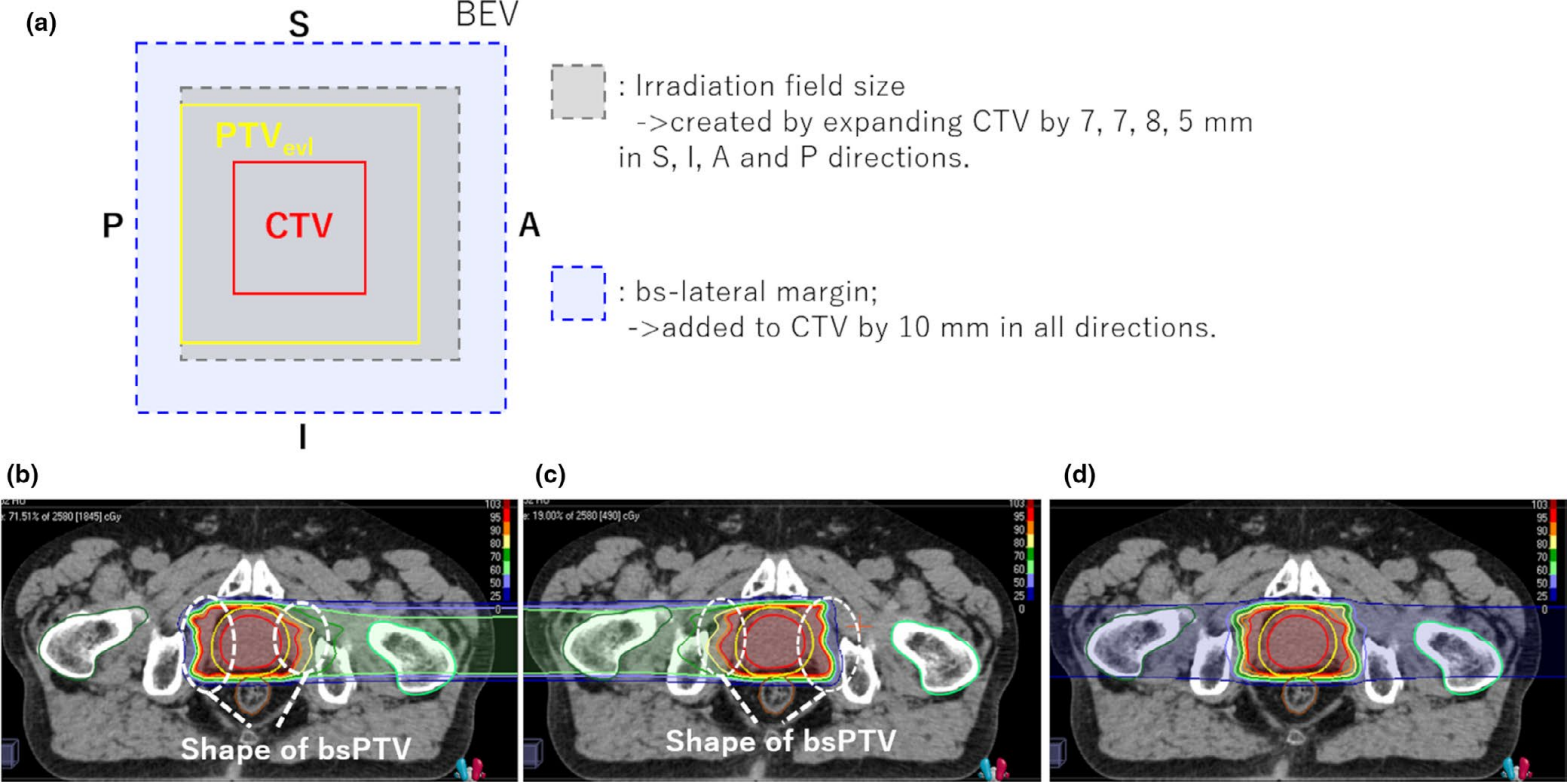
(a) Relationship between the margin of PTVevl, bsPTV, and irradiation field size. (b–d) show the representative example of dose distribution created by bsPTV. The shape of dose distribution at distal and proximal ends is distorted (inside the areas marked in white dashed line). Region of interests in (b–d) show CTV (red color) and PTV_evl_ (yellow color). bsPTV, beam‐specific Planning Target Volume

In the process of treatment planning, CT values in the delineated regions of the GAs and artifact around them are converted to 40 HU, which is equivalent to CT values of the prostate, because of negligible effect of Gas on dose distributions. Moreover, 10 mm of bs‐lateral margins (AP and SI margins in Table 1) reflects to the internal motion of prostate (>8 mm). In order to satisfy the clinical goal for PTV_evl_, whose margins are 8, 5, and 5 mm in LR, SI, and AP directions, respectively, the irradiation field for the CTV is set to 5, 8, 7, and 7 mm in posterior, anterior, inferior, and superior directions, specified by a virtual collimator in VQA (Figure 5a). The margins at QST hospital are set to 10, 6, and 5 mm in LR and anterior, SI, and posterior directions, respectively^29^ and the margin in posterior direction is changed from 5 to 3 mm in the last four fractions through treatment to reduce rectum dose.^4^ On the other hand, treatment with fiducial markers can provide a smaller irradiation field and the margins are constant during treatment. As Figure 6b–d show, the shape of the dose distribution created by treatment plans using bsPTV is distorted, resulting in a robust dose distribution against structural changes along the beam path caused by marker matching.

Fiducial marker‐based position verification gives anatomical changes along the beam path, which greatly differs from that of only bone matching, and the changes can be compensated by bsPTV treatment plans. The fiducial markers provide the accurate detection of the target and dose delivery to the target more accurately. Moreover, it is assumed that the marker matching ensures the reproducibility of the target and organs at risk (OAR) doses during treatment, leading to preventing unintended high doses to OAR, especially the rectum.

In clinical practice, if the amount of rigid translation to the marker is >5 mm twice in a row before six fractions, CT simulations are repeated, and a new treatment plan is created depending on the situation. The process of creating treatment plans presented in our study can be a valuable reference for treating prostate cancers with image‐guided CIRT using fiducial markers or in‐room CT.

## 5 CONCLUSION

We found that the dose perturbation due to fiducial markers is small in CIRT and can be ignored in the treatment planning. The margins of bsPTV were determined based on the results of target motions. In Osaka HIMAK, the treatment plans based on bsPTV are provided to treat prostate cancers with carbon ions using fiducial markers.

## CONFLICT OF INTEREST

The authors have no conflict of interest to disclose.

## AUTHOR CONTRIBUTION

T. Tsubouchi, N. Hamatani, M. Takashina, M. Yagi, M. Chatani, J. Mizoe, and T. Kanai were involved in study design and data interpretation. T. Tsubouchi, Y. Wakisaka, A. Ogawa, A. Terasawa, and K. Shimazaki were involved in the data analysis and experiments. All authors critically revised the report, commented on drafts of the manuscript, and approved the final report.

## Data Availability

The data that support the findings of this study are available from the corresponding author, Toshiro Tsubouchi, upon reasonable request.
